# Associations between Sleep Duration and Overweight/Obesity: Results from 66,817 Chinese Adolescents

**DOI:** 10.1038/srep16686

**Published:** 2015-11-16

**Authors:** Jie Wu, Hong Wu, Juan Wang, Lan Guo, Xueqing Deng, Ciyong Lu

**Affiliations:** 1 School of Public Health, Sun Yat-sen University, Department of Medical Statistics and Epidemiology Guangzhou, 510080 China; 2 UCLA Fielding School of Public Health, University of California Los Angeles, Department of Epidemiology, Los Angeles, 90066 United States of America

## Abstract

The findings about the shapes of associations between sleep duration and overweight/obesity in adolescents were largely inconsistent in the existing literature. We examined the functional forms of the associations between sleep duration and overweight/obesity in 66,817 Chinese adolescents by modelling sleep duration categorically and continuously. The adjusted ORs (95% CI) of overweight (with 7.0–8.9 h of sleep being considered the reference group) for subjects reporting <5.0 hours, 5.0–6.9 hours and ≥9.0 hours of sleep were 1.26 (1.05–1.51), 1.06 (1.00–1.11) and 1.27 (1.14–1.42), respectively. The adjusted ORs (95% CI) of obesity (with 7.0–8.9 h of daily sleep being considered as the reference group) for adolescents reporting <5.0 hours, 5.0–6.9 hours and ≥9.0 hours of sleep were 1.24 (0.97–1.57), 0.94 (0.87–1.01) and 1.42 (1.24–1.63), respectively. Continuous splines regressions support non-linear U shape associations between sleep duration and overweight/obesity, with the bottom at around 7.0–8.0 hours sleep (overweight: likelihood ratio = 32.7 p < 0.01; obesity: likelihood ratio = 40.4 p < 0.01). U-shape associations were found between sleep duration and overweight/obesity in Chinese adolescents and an optimal sleep duration of 7.0–8.0 hours sleep may prevent overweight/obesity.

The proportion of adolescents who are overweight and obese has increased dramatically in developed and some developing countries during the past few decades^1^. The prevention of overweight/obesity conventionally focuses on nutrition and physical activity^2^,^3^,^4^. However, most of such interventions suggested limited effectiveness^5^,^6^. Recently, the concentration has been shifted to sleep, a newly recognized modifiable factor. Previous epidemiologic studies in Western countries have obtained evidence of associations between sleep duration and overweight/obesity in adolescents^7^,^8^,^9^,^10^,^11^,^12^. However, little is known in non-Western settings, in which the social patterning of both sleep and overweight/obesity of adolescents could be divergent^13^,^14^. Moreover, the findings from the literature are largely inconsistent. For instance, several studies of adolescents have found negative linear associations between sleep duration and overweight/obesity^8^,^9^,^10^,^11^, while others have spotted U-shape associations^12^,^15^ or even null association^16^,^17^. Most previous studies categorized sleep duration, which may compromise the power to delineate the shape of associations^18^. To our limited knowledge, no previous study has used restricted cubic splines method, a more flexible and efficient method, to evaluate the potential non-linear associations between sleep duration and overweight/obesity among adolescents whether in Western settings or in non-Western settings.

In addition, there was no previous study, whether in adults or in children and adolescents, has investigated the joint associations of physical activity, sleep duration and overweight/obesity. Like sleep, physical activity is fundamental activity of daily life and is closely involved in general health. Low physical activity is an important predictor of overweight/obesity because it decreases energy expenditure^19^, and some proposed that this relationship may partially account for the observed health effects of sleep^20^. However, previous studies on the associations between sleep duration and overweight/obesity have adjusted for physical activity, a confounder considered to be in the associations of sleep duration and overweight/obesity. No study has been done to explore the possible effect modifier role of physical activity in associations between sleep duration and overweight/obesity. Understanding these complex joint associations is crucial for understanding and tackling overweight/obesity in adolescents. Because this may shed light on the mechanisms through which sleep exerts its health impact and to examine whether physical activity and sleep have a combined associations with adolescents’ overweight/obesity.

Against the above background, we conducted a study of the associations between sleep duration and overweight/obesity in a large sample of Chinese 10 to 18 years old adolescents. The primary aim of our study is to better delineate the functional forms of the associations between sleep duration and overweight/obesity in adolescents by modeling sleep duration categorically and linearly. The secondary objective is to explore whether the associations between sleep duration and overweight/obesity would be modified and to what extent by physical activity as well as gender.

## Results

After excluding missing data on sleep duration, height and weight (n = 16,459), 66,817 participants were finally included in this study. Those who were excluded were similar to the sample we analyzed in terms of demographics and lifestyles (data not shown). The participants’ characteristics are summarized in Table 1. Compared with those sleeping 7.0–8.9 hours/day, subjects sleeping <5.0 hour/day were more likely to be high school students, have parents with college degree, have time spent playing internet and video games ≥2 hours/day, have home work ≥3 hours/day, be in single family, use tobacco/alcohol and report depression and anxiety. Compared with those sleeping 7.0–8.9 hours, longer sleeper (≥9.0 hours/day) were more likely to be males, be junior high school students. Longer sleeper were less likely to report depression and anxiety. With respect to overweight and obesity, both short sleeper (<5.0 hours/day) and longer sleeper (≥9.0 hours/day) were more likely to report overweight and obesity, compared to those sleeping 7.0–8.9 hours/day.

**Figure 1:**
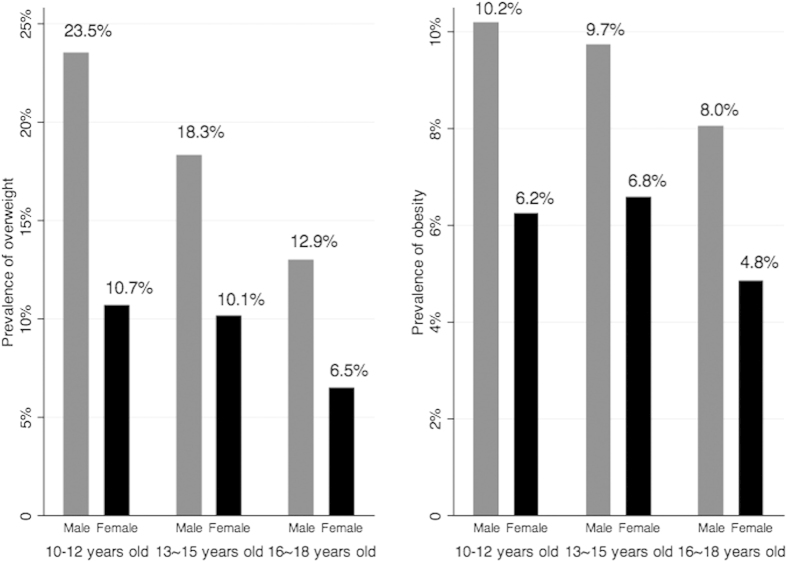
Age-and gender-specific prevalence of overweight and obesity.

Figure 1 presents age-and gender-specific prevalence of overweight/obesity. With regard to overweight, males were generally more likely to report overweight than females in all three age groups. There was a significant decreasing trend of prevalence of overweight with increasing age in both genders (males: p_trend_ < 0.01; females: p_trend_ < 0.01). In terms of obesity, similar patterns were found: males were generally more likely to report obesity than females in all three age groups. There was a significant decreasing trend of prevalence of obesity with increasing age in males (p_trend_ < 0.01), however no significant decreasing trend of prevalence of obesity with increasing age in females was identified.

Table 2 presents the associations between sleep duration and overweight/obesity. A U-shape association between sleep duration and overweight was found. The demographics-adjusted ORs (95% CI) of overweight (with 7.0–8.9 h of sleep being considered the reference group) for individual reporting <5.0 hours, 5.0–6.9 hours and ≥9.0 hours of sleep were 1.27 (1.07–1.51), 1.04 (0.99–1.09) and 1.28 (1.15–1.42), respectively. The results were similar with the results in model 2 adjusting for lifestyle variables and that in model 3 adjusting for mental health indicators. In terms of obesity, a similar U-shape pattern was found. The demographics-adjusted ORs (95% CI) of obesity (with 7.0–8.9 h of daily sleep being considered the reference group) for individual reporting <5.0 hours, 5.0–6.9 hours and ≥9.0 hours of sleep were 1.26 (1.01–1.59), 0.91 (0.86–0.98) and 1.45 (1.27–1.65), respectively. After adjusting for lifestyle variables and mental health indicators, the associations attenuate slightly for both short sleep (<5.0 hours/day) and longer sleep (≥9.0 hours/day), and the association for short sleep (<5.0 hours/day) become marginally insignificant.

In the joint analysis of sleep duration, gender and the odds of overweight/obesity (Table 3), Male participants sleeping <5.0 hours/day had the highest odds for overweight, compared with those male students sleeping 7.0 to 8.9 hours. Male participants sleeping ≥9.0 hours/day had the highest risk for obesity, compared with those boys sleeping 7.0 to 8.9 hours. However, we did not observe significant effect modification of gender for the above two sleep categories. Instead, there was a significant effect modification of gender in 5.0–6.9 hours sleep category. The ratio of ORs was 1.13 (1.03–1.25), which indicates a positive effect modification of gender on the odds ratio scale. The RERI was 0.08 (95% CI: 0.01–0.16), suggesting there is positive effect modification of gender on the additive scale. Male participants sleeping ≥9.0 hours/day had the highest odds for obesity, compared with those male students sleeping 7.0 to 8.9 hours. There was a significant additive effect modification of gender in 5.0–6.9 hours sleep category for obesity. The multiplicative effect modification of gender in 5.0–6.9 hours sleep category for obesity was marginally significant.

Table 4 shows the effect modification of physical activities on the associations between sleep duration and overweight/obesity. Participants with low physical activity sleeping ≥9.0 hours/day had the highest odds for both overweight and obesity, compared to students with low physical activity sleeping ≥9.0 hours/day. However, there was no significant effect modification of physical activity on multiplicative and additive scale.

**Figure 2:**
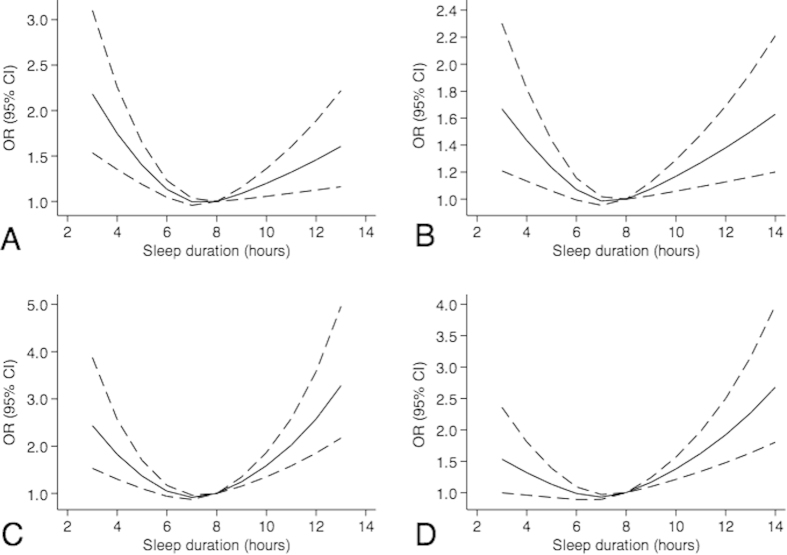
Non-linear regression curves of associations between sleep duration and overweight/obesity among boys and girls. (**A**) overweight among girls; (**B**) overweight among boys; (**C**) obesity among girls; (**D**) obesity among boys. All analyses were adjusted for age, whether from single family, parental education, parental smoking, Physical activity, internet and video games, homework, tobacco and alcohol use, depression and anxiety.

Based on the likelihood ratio test between the model with restricted cubic splines and the model without the splines, there were significant non-linear relationships between sleep duration and overweight/obesity (overweight: likelihood ratio = 32.7, p < 0.01; obesity: likelihood ratio = 40.4, p < 0.01). To illustrate these associations, we graphed the ORs and 95% CI from models using restricted cubic splines for females and males (Fig. 2). Results indicated that the shapes of the associations were similar for overweight/obesity, an U-shape with the bottom at around 7.0–8.0 hours sleep. However, for obesity, a steeper slope was observed for long sleep duration.

## Discussion

In the present study, we found clear U-shape associations between sleep duration and overweight/obesity in Chinese adolescents population. We also found gender and sleep duration may have a synergistic association with overweight/obesity.

Our result that short sleep duration was associated with overweight/obesity are in line with an earlier cross-sectional study, it was estimated that for each hour sleep lost, the odds of being obese increased in adolescents by 80%^11^. Furthermore, in logistic model with restricted cubic splines, we found the associations were stronger with the decrease of sleep duration. This finding is in accordance with a prior study, in which they observed a “dose–response” relationship between sleep and overweight^21^, with odds ratios of overweight increasing with decreasing sleep duration (<5.0 hours/day, 5.0–6.9 hours/day, and 7.0–8.9 hours/day compared with students sleeping ≥9.0 hours/day).

Notably, there was substantial heterogeneity across studies about the shape of the associations between sleep duration and overweight/obesity. Among Norwegian children aged 10–12 years old, Danielson, *et al.*^15^ observed U-shape associations between sleep duration and overweight/obesity as well as BMI. Similarly, Sivertsen *et al.*^12^ also observed a U-shape association between sleep duration and BMI categories. However, a gender-stratified analysis supported this U-shape association for girls only. There were also many studies revealing a different picture. Among a large and ethnic diverse US adolescents sample, Reither and colleagues^22^ rejected a non-linear association and suggested a negative linear association between sleep duration and body mass. Bawazeer *et al.* only observed^7^, in 5877 children and adolescents aged 10–19 years, an association between short sleep duration and obesity but there was no association observed between long sleep duration and obesity. In our study, short sleep duration and long sleep duration were both associated with overweight/obesity, and the statistical tests of restricted cubic splines also supported U-shape associations between sleep duration and overweight/obesity. These discrepancies in results between studies might be driven by many reasons including different study locations, study design, different age of sample, varied exposure categories and different confounder adjusted. Further large mete-analysis or pool analyses are warranted to explore the potential correlates of heterogeneity of findings existing in current literature.

In our study, we found that gender and sleep duration may have a synergistic association with overweight/obesity, the combination of being boys and sleeping 5.0–6.9 hours has stronger association with overweight/obesity than the sum of their individual associations with overweight/obesity, indicating boys may be more susceptible to the adverse effects associated with short sleep. Some previous studies also found sex differential associations between sleep and weight/obesity. For instances, a study in Japan^8^ indicated that boys may be at higher risk for having overweight/obesity when they had the same level of sleep duration with girls. Similarly, other studies found associations between short sleep and obesity only among boys 9,23. However, previous studies did not report the measures of this effect modification, which impedes the direct comparisons with our study.

In our study, we did not obtain evidence that physical activity may modify the associations between sleep duration and overweight/obesity. This finding highlights the importance of sleep as a health behavior distinct from physical activity, and indicates that physical activity may not neutralize the adverse effect associated with altered sleep. Therefore, even among physical active adolescents, improving sleep hygiene may show additional benefits in terms of preventing overweight/obesity.

One of interesting findings is that the associations we observed between sleep duration and overweight/obesity remained after adjusting for depression and anxiety. It has been proposed that mental health, such as depression, decreases people’s physical activity, and alters sleep, which could confound the associations between sleep duration and overweight/obesity^24^. However, this was not supported by our study. While the possibility of residual confounding cannot be excluded, our finding may further underscore the independent role of sleep in overweight and obesity.

Recent evidence suggests a biological plausibility of the relationship between short sleep duration and overweight/obesity. It was found that short sleep duration was associated with decreased leptin levels, increased ghrelin levels and increased hunger and appetite, which may possibly account for the increased BMI^25^,^26^. Alternatively, the associations between short sleep duration and overweight/obesity may be explained by short sleep disrupting circadian rhythms, which lead to abnormal timing of adipocyte differentiation and the release of adipokines^27^,^28^. A cross-sectional study supported that adolescents going to bed late were more likely to have higher BMIs, independent of sleep duration, compared with adolescents who go to bed early^29^. Finally, it is possible that sleep deprivation may be an epiphenomena for poorer health outcomes or lower quality of life rather than a casual factor for overweight/obesity^30^.

The mechanism of increased risk of overweight/obesity in long sleepers is nuclear. It has been postulated that in long sleepers, reduced energy expenditure due to increased time in bed may affect their obesity. In support, a study has shown that long sleepers exercise less^31^. Unfortunately, this theory may not explain the associations observed in our study. Our results indicated that the associations between long sleep duration and overweight/obesity still exist after controlling for physical activity. Another possible explanation is that adolescents with sleep disordered breathing (SDB), a medical condition associated with increased BMI, may spend more time in bed to reimburse fragmented sleep^25^.

Our study has several strengths. In the first place, our study used a large and randomly selected Chinese adolescents sample, which renders us sufficient statistical power to detect possible associations even after adjusting for a considerable of potential confounders. In the second place, unlike previous studies, our study modeled sleep duration continuously with restricted cubic splines, which prevents the loss of information when a continuous variable is divided into broad categories.

Our findings should also be treated with the following limitations. The main limitation of our study is a cross-sectional design, which only allows us to examine the still or cross-sectional associations rather than dynamic or longitudinal associations, which severely undermine our ability to make causal inferences. Another major flaw of our study is that we used self-reported data. Self-reported sleep duration may be systematically longer than actigraphy-measured sleep^32^, and self-reported BMI are likely to be underestimated. This systematic measurement error may directly lead to non-differential exposure and outcome misclassification that eventually bias the expected values of OR toward the null^33^,^34^, therefore the estimated ORs were conservative. In addition, the self-reported covariates, such as physical activity, time spent playing internet and video games, etc., may increase the possibility of residual confounding by those covariates. Furthermore, we only collected information for sleep duration on weekday. Therefore, if weekend sleep plays important role in adolescents’ energy balance, we may not able to adjust for it. Last but not least, Although our study adjusted a wide range of potential confounders, the estimated associations could still be confounded by unmeasured factors; for example, energy intake, a potential confounder, may confound the observed associations, although associations were similar between model without controlling for energy intake and model that controlled for energy intake^35^. Family history of obesity was also not adjusted in our study, which may positively confound the association between sleep duration and adolescent overweight/obesity. In addition, the interpretation of effect modification analysis depends on strong assumption that there is no unmeasured confounding, which is hardly to be true in practice. Therefore, the analyses of effect modification only suggest the heterogeneity of the associations between sleep and overweight/obesity.

Our study demonstrates U-shape associations between sleep duration and overweight/obesity. If our finding is confirmed by further study, then there may be an “optimal zone” of sleep duration for adolescents, outside of which harmful effects of sleeping may disturb energy balance. In addition, Because of the high prevalence of insufficient sleep duration in adolescents, these findings highlight the urgent needs to address sleep behaviors in adolescents as part of routine health care.

## Methods

### Participants

Our study was a cross-sectional and province-wide representative survey of Chinese students in grades 7–12. We employed stratified cluster multistage sampling to select the sample. The first stage sampling was the selection of primary sampling units (PSUs), which were all of the 21 administrative regions in Guangdong province, China. The second sampling stage was the selection of schools within the PSUs. All eligible schools in each administrative region were stratified by type of school (junior high school, high school, and vocational school), size of school (small, middle, and large) and academic performance (key school and regular school). The bureau of education in each administrative region provided us with a list of all schools serving students in grades 7–12 as well as the information required for stratification. In each administrative region, 8–12 schools consisting of 3–4 junior high schools, 3–4 high schools and 2–4 vocational schools were randomly selected. A total of 291 schools were finally selected. The last stage of sampling was the selection of classes, which were stratified by grade. We randomly selected two classes in each grade. In each selected class, every student was invited to participate in our survey. Original data were collected from *N* = 83,276 students (reached sample *N* = 104,760, response rate 79%) with a mean age of 16.6 years, between September 2011 and January 2012.

The study protocol was reviewed and approved by the ethics committees of Sun Yat-sen University. The principals of the schools attended by the participants also reviewed and approved the study procedure. Oral informed consent was obtained from each participant. The methods were carried out in accordance with the approved guidelines.

### Measures

All students who consented to participate in the study were asked to complete self-administered surveys under exam-like conditions within one 45-minute class period in their own classroom. The students were required to complete their own questionnaires by themselves. If they had any questions, they could ask local health professionals present in the classroom. However, the teachers and school staff were asked to be absent during the survey.

### Assessment of sleep duration and physical activity

In our study, we specifically measured weekday sleep duration. Participants estimated what time they would go to bed and wake up on a normal weekday, and sleep duration (converted to hours) was then calculated from their estimates. Prior study has indicated that child-reported sleep durations are moderately to strongly correlated with sleep durations from actigraphy and sleep diaries^36^.

Participants were given a list of common physical activities such as bicycling, football, basketball, etc., and they were asked to indicate how often (never or occasionally, weekly but <1 hour/week, 1–3 hours/week, 4–7 hours/week and above) they had engaged in these activities during the past year. Physical activity was further dichotomized as 0 for never/occasionally or <1 hour/week and 1 for 1–3 hours/week, 4–7 hours/week and above.

### Assessment of Outcome

Students self-reported their height (in meters) and weight (in kg) with open-ended response questions. Body mass index (BMI) was calculated from these reported data. Previous studies suggested that subjective reports tend to underestimate true BMI and that the prevalence of overweight/obesity eventually tends to be under-reported^37^. Therefore, a method suggested by a prior study was used to correct the self-reported BMI^38^. The corrected BMI was then used to determine overweight and obesity. Overweight and obesity were defined by age- and sex-specific cut-off points that correspond to an adult BMI of 25 and 30, as developed by the Childhood Obesity Working Group of the International Obesity Task Force (IOTF)^39^.

### Covariates

We also collected information on a diversity of covariates, including age, gender, coming from single family (yes or no), parents’ education level (both had college degree, one of them had college degree or neither had a college degree) as a proxy for socioeconomic status (SES), parental smoking (at least one of them smoke or neither of them smoke). We also asked about lifestyle factors, such as time spent on homework (≥3 h or <3 h), time spent playing internet and video games (≥2 h or <2 h), tobacco (smoked in past 30 days or not) and alcohol consumption (used alcohol in past 30 days or not) consumption. In addition, As indicators of depression and anxiety, we asked the following two questions, adopted from the Schools Sleep Habits Survey^40^: ‘During the past two weeks, how often were you bothered by the following?’ (1) Feeling unhappy, sad or depressed?; and (2) feeling nervous or tense? (Not at all, somewhat, or a lot).

### Statistical analysis

Subjects’ demographic-characteristics, life-style habits, sleep duration and overweight/obesity were summarized with means and standard deviations (SDs) for continuous variables, and frequencies and proportions were described for categorical variables. We used sleep duration as both a categorical variable and a continuous variable to estimate its association with overweight/obesity. Sleep duration was categorized as <5.0 hours/day, 5.0-6.9 hours/day, 7.0–8.9 hours/day and ≥9.0 hours/day.

Multivariate logistic regression analyses were performed to estimate the associations between categories of sleep duration and overweight/obesity, using 7.0–8.9 hours/day of sleep as the reference category. Three sets of logistic regression model were used to evaluate the associations. The initial logistic regression model was adjusted for subjects’ demographic characteristics, including age, gender, parental education, parental smoking and coming from single family (model 1). The subsequent model was additionally adjusted for participants’ lifestyle variables including physical activity, time spent playing internet and video games, homework, smoking and alcohol use (model 2). In model 3, we further adjusted for mental health indicators such as depression and anxiety. All potential confounders were selected as a priori, based on scientific evidence.

To evaluate whether there were multiplicative and additive effect modifications of gender and physical activity, we conducted a subgroup analysis of sleep and overweight/obesity stratified according to these 2 variables. As suggested by Knol *et al.*^41^, we used the ratio of odds ratios (ORs) and its 95% Confidence Intervals (CIs) to measure the effect modification on a multiplicative scale. To evaluate the additive effect modification, we calculated the relative excess risk due to interaction (RERI) and its 95% CI, which is considered to be the standard measure for effect modification on an additive scale.

To explore the possible non-linear shape of the OR function, we fitted a logistic regression model with restricted cubic splines in which sleep duration was treated as continuous variable^42^,^43^. We specified 3 knots at 5.0, 7.0 and 9.0 hours of sleep, corresponding to the categories used in logistic regressions in which sleep duration was used as a categorical variable. The covariates adjusted in the logistic regression model with restricted cubic splines were the same as the covariates adjusted in model 3. ORs for overweight and obesity were assessed by comparison to subjects who reported sleep duration of 8.0 hours. Non-linearity was tested using the likelihood ratio test, comparing the model with only linear term to the model with the linear term and restricted cubic splines.

Because of the multi-stage sampling procedure, all ORs and 95% CIs were weighted by the probability of selection, and all frequencies were weighted with Taylor series linearization to adjust for variations in sample selection probability^44^. For this part of the analysis, svy: table, svy:mean and svy: logistic of STATA, version 12.1, software (StataCorp LP, College Station, Texas) were used. All statistical tests were 2-tailed, and probability values <0.05 were considered statistically significant.

## Additional Information

**How to cite this article**: Wu, J. *et al.* Associations between Sleep Duration and Overweight/Obesity: Results from 66,817 Chinese Adolescents. *Sci. Rep.* **5**, 16686; doi: 10.1038/srep16686 (2015).

## Figures and Tables

**Table 1:** Characteristics of participants by sleep duration category (N = 66,817).

Characteristic	Sleep Duration, hours/day											
	<5.0			5.0 ~ 6.9			7.0 ~ 8.9			≥9.0		
	*N*	%wt	Mean (SD)	*N*	%wt	Mean (SD)	*N*	%wt	Mean (SD)	*N*	%wt	Mean (SD)
Female sex	487	47.0		14,263	53.5		20,993	50.1		983	37.2	
Age, years			16.2 (1.5)			16.4 (1.4)			15.5 (1.7)			14.9 (1.5)
Grade												
Junior high school^§^	418	42.5		8,530	33.7		23,150	58.1		1,832	74.7	
High school & vocational school^¶^	533	57.5		16,053	66.3		15,678	41.9		623	26.3	
Parental smoking	496	54.4		12,420	52.3		19,340	51.8		1,237	53.0	
Parents highest education at college level												
Both had college degree	82	8.7		1,029	4.4		1,809	4.8		115	5.1	
Only one of them had college degree	101	11.0		1,855	7.8		2,821	7.5		140	5.9	
None of them had college degree	756	80.3		21,433	87.8		33,669	87.7		2,158	89.0	
Single family	87	9.6		1,302	5.4		1,617	4.3		100	4.3	
Sleep duration, hours			4.5 (0.7)			6.7 (0.4)			8.0 (0.5)			9.8 (0.6)
Reported BMI^&^			21.1 (6.3)			20.3 (5.1)			20.0 (5.3)			20.3 (5.9)
Corrected BMI^&,$^			22.1 (6.6)			21.5 (5.5)			21.7 (6.2)			21.7 (6.2)
Overweight	179	23.6		3,498	16.4		6,430	18.8		559	26.8	
Obesity	94	16.0		1,602	11.2		3,081	13.2		302	18.8	
Physical activity	308	32.3		7,757	31.3		9,552	24.7		528	27.2	
Computer and video games ≥2 hours/day	309	34.0		3,218	13.8		3,521	9.6		187	8.3	
Home work ≥3 hours/day	362	38.3		7,695	31.1		5,191	13.6		205	8.5	
Smoking	156	18.0		1,209	5.5		1,405	4.1		129	6.2	
Alcohol	360	38.9		4,880	21.1		6,168	16.9		387	16.9	
Depression	616	64.7		14,042	57.2		15,708	40.8		709	29.5	
Anxiety	522	55.5		11,431	46.8		14,240	37.0		706	29.2	

Abrreviations: BMI; Bady Mass Index; SD: Standard Deviation.

^§^Grades 7–9.

^¶^Grades 10–12; ^&^Weight (kg)/height (m2); ^$^BMI after correction.

**Table 2:** Associations between sleep duration and overweight/obesity among adolecents (N= 66,817).

Models	Sleep Duration, hours/day											
	<5.0			5.0–6.9			7.0–8.9			≥9.0		
	Cases/controls	OR	95% CI	Cases/controls	OR	95% CI	Cases/controls	OR	95% CI	Cases/controls	OR	95% CI
Overweight	179/772			34,98/21,085			6,430/32,398			559/1,896		
Model 1		1.27	1.07–1.51		1.04	0.99–1.09		1.00	Referent		1.28	1.15–1.42
Model 2		1.25	1.04–1.49		1.05	1.00–1.10		1.00	Referent		1.28	1.15–1.42
Model 3		1.26	1.05–1.51		1.06	1.00–1.11		1.00	Referent		1.27	1.14–1.42
Obesity	94/857			1,602/22,981			3,081/35,747			302/2,153		
Model 1		1.26	1.01–1.59		0.91	0.86–0.98		1.00	Referent		1.45	1.27–1.65
Model 2		1.21	0.95–1.54		0.93	0.87–1.00		1.00	Referent		1.42	1.24–1.63
Model 3		1.24	0.97–1.57		0.94	0.87–1.01		1.00	Referent		1.42	1.24–1.63

Abrreviations: OR: Odds Ratio; 95% CI: 95% Confidence Interval.

Notes: Cases: the number of participants who were overweight or obese; controls: the number of participants who were not overweight or obese.

Model 1: adjusted for age, gender, coming from single family, parental education and parental smoking.

Model 2: adjusted for covariates in model 1 + physical activity, internet and vedio games, homework, tobacco and alcohol use.

Model 3: adjusted for covariates in model 2 + depression and anxiety indicators.

**Table 3:** Effect modification of gender on associations between sleep duration and overweight/obesity (N = 66,817).

Variables	Sleep Duration, hours/day							
	<5.0		5.0–6.9		7.0–8.9		≥9.0	
	Cases/controls	OR (95% CI)	Cases/controls	OR (95% CI)	Cases/controls	OR (95% CI)	Cases/controls	OR (95% CI)
Overweight								
** **Male	101/363	1.24 (0.98–1.58)	1,816/8,504	0.99 (0.93–1.06)	3,804/14,031	1.00	390/1,082	1.23 (1.08–1.41)
** **Female	78/409	0.73 (0.56–0.96)	1,682/12,581	0.64 (0.60–0.69)	2,626/18,367	0.57 (0.53–0.60)	169/814	0.76 (0.63–0.91)
** **Ratio of ORs^¶^	1.04 (0.72–1.49)		1.13 (1.03–1.25)				1.08 (0.86–1.36)	
** **RERI^§^	−0.08 (−0.38–0.23)		0.08 (0.01–0.16)				−0.04 (−0.18–0.10)	
Obesity								
** **Male	50/414	1.22 (0.89–1.68)	822/9,498	0.89 (0.81–0.98)	1,773/16,062	1.00	203/1,269	1.33 (1.12–1.59)
** **Female	44/443	0.80 (0.55–1.15)	780/13,483	0.63 (0.57–0.70)	1,308/13,483	0.64 (0.59–0.69)	99/884	1.01 (0.80–1.27)
** **Ratio of ORs^¶^	1.02 (0.63–1.65)		1.11 (0.97–1.26)				1.18 (0.89–1.57)	
** **RERI^§^	−0.06 (−0.47–0.34)		0.26 (0.22–0.31)				0.04 (−0.26–0.33)	

Abrreviations: OR: Odds Ratio; 95% CI: 95% Confidence Interval; RERI: Relative Excess Risk due to Interaction.

Notes: Cases: the number of participants who were overweight or obese; controls: the number of participants who were not overweight or obese.

^¶^effect modification on multiplicative scale. ^§^effect modification on additive scale.

**Table 4:** Effect modification of physical activity on associations between sleep duration and overweight/obesity (N = 66,817).

Variables	Sleep Duration, hours/day							
	<5.0		5.0–6.9		7.0–8.9		≥9.0	
	Cases/controls	OR (95% CI)	Cases/controls	OR (95% CI)	Cases/controls	OR (95% CI)	Cases/controls	OR (95% CI)
Overweight								
PA = 0	119/507	1.25 (1.01–1.55)	2,495/13,951	1.08 (1.02–1.15)	4,865/23,595	1.00	441/1,442	1.30 (1.15 –1.47)
PA = 1	55/253	1.29 (0.94–1.77)	944/6,813	0.99 (0.91–1.08)	1,411/8,141	1.00 (0.93–1.07)	102/426	1.14 (0.90–1.46)
Ratio of ORs^¶^	1.03 (0.70–1.51)			0.92 (0.82–1.02)			0.87 (0.67–1.15)	
RERI^§^	0.04 (−0.46–0.53)			−0.09 (−0.20–0.02)			−0.16 (−0.48–0.15)	
Obesity								
PA = 0	62/564	1.32 (1.00–1.76)	110/15,336	0.96 (0.88–1.04)	2,286/26,174	1.00	231/1,634	1.45 (1.24–1.70)
PA = 1	28/280	1.12 (0.72–1.75)	464/7,293	0.94 (0.84–1.05)	721/8,831	1.07 (0.97–1.17)	60/468	1.40 (1.03–1.89)
Ratio of ORs^¶^	0.79 (0.47–1.34)		0.92 (0.79–1.06)				0.90 (0.64–1.27)	
RERI^§^	−0.27 (−0.86–0.32)		−0.09 (−0.23–0.05)				−0.12 (−0.61–0.36)	

Abrreviations: PA: Physical Activity; OR: Odds Ratio; 95% CI: 95% Confidence Interval; RERI: Relative Excess Risk due to Interaction.

Notes: Cases: the number of participants who were overweight or obese; controls: the number of participants who were not overweight or obese.

^¶^effect modification on multiplicative scale.

^§^effect modification on additive scale.
